# A Rapid and Quantitative Flow Cytometry Method for the Analysis of Membrane Disruptive Antimicrobial Activity

**DOI:** 10.1371/journal.pone.0151694

**Published:** 2016-03-17

**Authors:** Neil M. O’Brien-Simpson, Namfon Pantarat, Troy J. Attard, Katrina A. Walsh, Eric C. Reynolds

**Affiliations:** Oral Health Cooperative Research Centre, Melbourne Dental School, Bio21 Institute, The University of Melbourne, Melbourne, Australia; Faculdade de Medicina da Universidade de Lisboa, PORTUGAL

## Abstract

We describe a microbial flow cytometry method that quantifies within 3 hours antimicrobial peptide (AMP) activity, termed Minimum Membrane Disruptive Concentration (MDC). Increasing peptide concentration positively correlates with the extent of bacterial membrane disruption and the calculated MDC is equivalent to its MBC. The activity of AMPs representing three different membranolytic modes of action could be determined for a range of Gram positive and negative bacteria, including the ESKAPE pathogens, *E*. *coli* and MRSA. By using the MDC50 concentration of the parent AMP, the method provides high-throughput, quantitative screening of AMP analogues. A unique feature of the MDC assay is that it directly measures peptide/bacteria interactions and lysed cell numbers rather than bacteria survival as with MIC and MBC assays. With the threat of multi-drug resistant bacteria, this high-throughput MDC assay has the potential to aid in the development of novel antimicrobials that target bacteria with improved efficacy.

## Introduction

A recent report from the World Health Organisation has highlighted that antibiotic and multi-drug resistant bacteria are a major and growing issue facing public health worldwide and that “fostering innovation and research and development of new tools” is vital in tackling this problem [1]. It is now recognised that the rates and severity of infections caused by antibiotic and multi-drug resistant bacteria are increasing year by year and are becoming harder and more complicated to treat and manage [1, 2]. A Centres for Disease Control report has estimated that of the 2 million reported U.S. hospital infections 70% are caused by antibiotic resistant bacteria leading to 44,000 deaths per year [3]. The majority of these antibiotic resistant bacterial infections are principally caused by a small number of species; *Enterococcus faecium*, *Staphylococcus aureus*, *Klebsiella pneumoniae*, *Acinetobacter baumannii*, *Pseudomonas aeruginosa*, and Enterobacter species, collectively termed the ESKAPE pathogens [4]. Initially the antibiotic resistant strains of these bacteria were restricted to nosocomial infections, however, a higher prevalence of antibiotic resistant infections is now emerging among community-acquired infections [5]. Of the ESKAPE pathogens *S*. *aureus* has received considerable attention as infections have become increasingly unresponsive to first-line antibiotic therapies and the frequency of methicillin resistance among *S*. *aureus* (MRSA) strains now ranges from 33–55% in U.S. hospitals, 20% in European hospitals, 38–54% in Japanese hospitals and in 2005 was reported to be 32% in Australian hospitals [6–8]. In 2005 the number of deaths in the U.S. attributed to MRSA infections was reported to be 18,650 which was higher than the reported number of deaths (17,000) attributed to HIV [9]. Despite the obvious need for new antibiotics, the major pharmaceutical companies have reduced funding in this area, conversely, increased academic research has led to significant developments in new antibiotic discovery platforms [10].

A new class of antimicrobial agent that has received considerable interest is antimicrobial peptides (AMPs) and hundreds of peptides with broad-spectrum and potent antimicrobial activity have been isolated from single celled organisms, invertebrates and vertebrates [11]. The great interest in AMPs is due to their mode of action in killing microbes, as it is distinct from conventional antibiotics and does not readily induce resistance [12]. While some AMPs have an intracellular mode of killing bacteria, the majority of AMPs to date act on the cytoplasmic membrane causing disruption and permeation of the membrane leading to cell death [11]. In general, AMPs are amphipathic and are classified according to their composition and secondary structure and typically have a more ordered structure when bound to a lipid membrane [11, 13]. On binding to a bacterial cell AMPs cause stretching/thinning of the cytoplacmic membrane and at a certain concentration, “threshold point”, the peptides insert into the membrane causing disruption or pore formation [13]. The extent of the membrane thinning is peptide sequence specific and is directly proportional to the peptide concentration [13]. Three broadly defined models have been proposed to explain membrane disruption and permeabilization by AMPs, these being the ‘barrel-stave’ model, the ‘toroidal pore’ model and the ‘carpet’ or ‘micelle’ model (reviewed in [11]). Essentially, each mechanism results in uncontrolled movement of ions and molecules into and out of the bacterial cell, leading to cell death.

The major advantages of AMPs are their rapid action and broad spectrum of activity, their ease of analogue synthesis and their low rate in selecting resistance compared with traditional antibiotics. Despite their natural origin, the few AMP candidates that have so far entered clinical trials have all been modified by rational or semi-rational chemical approaches to optimise efficacy, potency and specificity [14, 15]. Using these rational or semi-rational approaches in AMP development a large range of peptide analogues can be generated using standard solid phase peptide synthesis techniques. A “bottleneck” in AMP development is in determining the activity of these analogues against different bacteria, as the standard methods require a period of growth following incubation with the AMP.

The current approach in determining AMP activity (Minimum Inhibitory Concentration (MIC) and/or Minimum Bactericidal Concentration (MBC)) uses bacterial growth assays. These assays are relatively straightforward and have evolved into a 96 well plate format as a turbidity/microdilution assay [16]. Although these assays are quantitative and are used to directly compare AMPs and their analogues, they represent a severe bottleneck in the AMP screening process. Even in a 96 well format, only a few AMPs can be evaluated at a time. The method relies on bacterial growth and even if a fast growing species like *Escherichia coli* is used, the MIC assay takes around a day to complete and a further day is required for an MBC assay. However, most of the pathogen targets have much longer mean generation times than *E*. *coli* [17]. Further, many pathogenic bacteria are obligate anaerobes which require complex growth media and an anaerobic environment for growth. Since turbidometric assays for MIC and MBC determination is dependent on acquiring optimal growth conditions for the bacteria to be tested in solution media in a plate format, which is not always straight forward, the more labour intensive and time consuming colony forming unit (CFU) agar assays are used to determine MBC [18–21].

To overcome the limitations of the MIC and MBC assays several high throughput screening (HTS) systems have been developed. These include a range of computational in silico systems, cell-based *in vivo* systems and combinatorial chemistry approaches [22, 23]. A common feature of these methods is that thousands of potential antimicrobial agents are either designed and tested in silico or produced using combinatorial chemistries and screened on agar plates. Although these approaches have had their successes [22] there are drawbacks in their application; they are typically non-quantitative, do not discriminate between bacteriostatic and bactericidal AMPs and are difficult to modify for screening analogues of a target AMP which requires quantitative assays.

As outlined above, the current antimicrobial activity assays are labour-intensive and time consuming, allow only a few AMPs or bacteria to be screened at a time or are non-quantitative. Thus, there is an urgent requirement for a straightforward, quantitative high-throughput screening method that is comparable to the standard MIC/MBC assays. The research described here directly addresses this issue and demonstrates that microbial flow cytometry can be used to quantitatively assess antimicrobial activities of several distinct AMPs in a straightforward and high throughput assay.

## Results

### Rationale and development of a rapid, quantitative antimicrobial flow cytometry method

**Fig 1:**
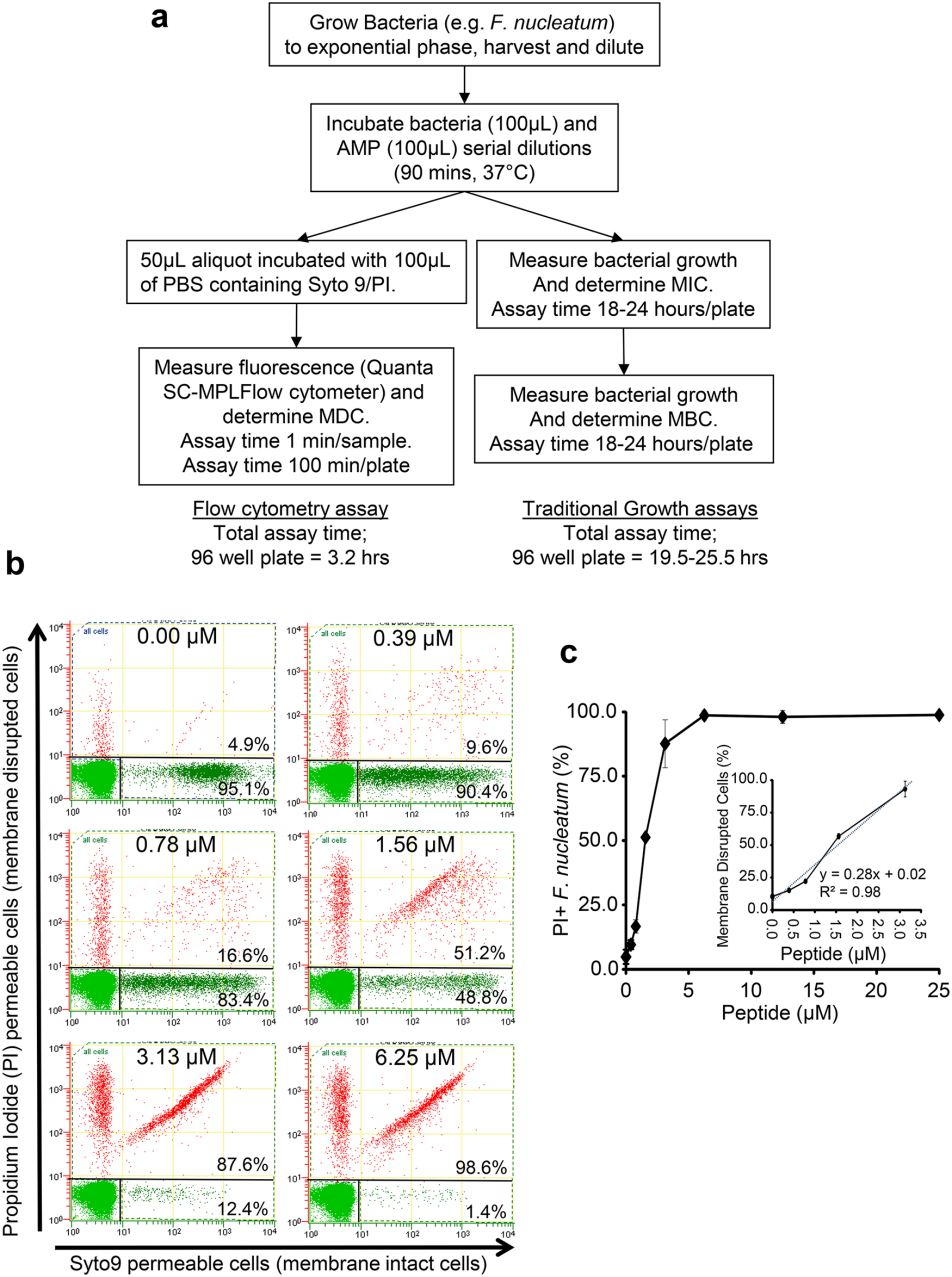
Increasing concentration of the antimicrobial peptide Melittin increases bacterial membrane permeability to propidium iodide. (a) Schematic representation of the work flow for determining MIC, MBC and MDC. (b) Flow cytometry dot blots of *F*. *nucleatum* incubated with increasing concentrations of Melittin and stained with Syto9 dye (membrane permeable) and propidium iodide dye (membrane impermeable). (c) Percent propidium iodide positive *F*. *nucleatum* cells correlates with increasing peptide concentration. Insert shows the reciprocal plot of percent membrane disrupted cells (PI+) and peptide concentration to determine MDC.

We reasoned that as AMPs disrupt the cytoplasmic membrane we should be able to monitor this disruption using membrane permeable/impermeable fluorescent intercalating DNA dyes that are used to determine the viability of a microbial culture. We initially synthesised melittin (peptide sequence shown in S1 Table) a well characterised, broad-spectrum and potent AMP to investigate whether viability dyes, Syto9 (membrane permeable) and propidium iodide (PI, membrane impermeable), could be used to determine AMP activity, against the melittin-susceptible bacterium *Fusobacterium nucleatum*. Initially, MIC and MBC (using standard CLSI methodology) of melittin for *F*. *nucleatum* were determined by harvesting late exponentially growing cells (to ensure ≥95% viable bacteria) and incubating (2.5 × 10^6^ cells/mL) with serial dilutions of melittin for 90 minutes, followed by bacterial growth assays (Fig 1a). Table 1 shows that *F*. *nucleatum* is highly melittin-sensitive with low MIC and MBC activities observed. Using the average MIC/MBC value of 4.0 μM we incubated *F*. *nucleatum* with melittin at 0.5× and 10× the MIC/MBC and then added varying concentrations of Syto 9/PI to evaluate whether peptide concentrations resulted in variant and observable membrane disrupted/non-disrupted cell populations. Staining cells with 0.1% v/v Syto 9 in 0.9% v/v saline (3.34mM stock solution) and 0.1% v/v PI in saline (50μg/mL stock solution) provided optimal concentrations that gave consistent and distinct cell populations; a membrane intact (Syto9+/PI-) and two membrane disrupted populations (Syto9+/PI+ and Syto9-/PI+; S1 Fig). At the optimal Syto 9/PI concentrations, incubating *F*. *nucleatum* with 2 μM and 40 μM melittin resulted in 48% and >97.5% of the bacteria being PI+ (i.e. Syto9+/PI+ and Syto9-/PI+), respectively (S1 Fig). Following from this we then incubated *F*. *nucleatum* with variant melittin concentrations and determined the proportion of membrane intact (Syto9+/PI-) and membrane disrupted (PI+ i.e. Syto9+/PI+ and Syto9-/PI+) populations. Fig 1b shows that increasing melittin concentration resulted in a corresponding increase in membrane disrupted cells. At melittin concentrations of 3.13 and 6.25 μM and higher; 87% and >98% of the bacteria were PI+, respectively, indicating that the melittin concentration that induces 98%+ membrane disrupted bacteria is between 3.13–6.25 μM (Fig 1c). Linear regression of the PI+ values for the peptide concentrations prior to full membrane disruption (% PI+ cells ≤ 99% and ≥ 1–5%) gave an R^2^ value of >0.9 and from extrapolation the minimum peptide concentration that induced 100% membrane disruption (MDC) could be determined (Fig 1c, insert). For melittin the MDC was 3.3±0.3 μM, which was in agreement with the melittin MIC and MBC values (Table 1). Using another lytic AMP, magainin II, the membrane disruption of *F*. *nucleatum* displayed a similar positive correlation with AMP concentration. The MDC obtained was 9.5±2.4 μM which was again in agreement with the MIC and MBC for that AMP (S2 Fig and S2 Table). Melittin and magainin II are known to have high and low activities against the Gram positive bacterium *Streptococcus mutans*, respectively. Table 1 and S2 Table, shows that the MDC assay discriminated between the two peptides where melittin was found to be 25 fold more potent than magainin II against *S*. *mutans* and that the MDC was comparable with the MIC and MBC of these peptides. Although the standard procedure is a 90 minute incubation of bacteria and AMP, we investigated whether the incubation time could be shortened. Table 1 shows that shortening the incubation time affected the MDC for *F*. *nucleatum* but not for *S*. *mutans* when incubated with melittin, indicating that the AMP/bacteria incubation could be shortened but this is AMP/species dependant.

**Table 1:** MIC and MBC of melittin for *F*. *nucleatum* and *S*. *mutans* and comparison with the membrane disrupting concentration (MDC) determined at different peptide/bacteria incubation times.

	MIC^a^^,^ ^b^ (μM)	MBC^a^^,^ ^b^ (μM)	AMP/Bacteria Incubation time MDC (μM)^a^			
Bacterium	90 min	90 min	90 min	60 min	30 min	15 min
*F*. *nucleatum*^c^	4.4 ± 0.3	3.8 ± 0.4	3.3 ± 0.3	3.7 ± 0.2	7.3 ± 0.7	9.6 ± 0.1
*S*. *mutans*^c^	6.1 ± 0.9	4.2 ± 2.4	3.5 ± 0.9	3.3 ± 0.1	3.1 ± 0.1	3.3 ± 0.4

^a^Activity expressed as μM is the average of 3 assays ± standard deviation.

^b^MIC and MBC determined following incubation of bacteria with peptide for 90 mins using standard protocols [24].

^c^Bacterial strains *F*. *nucleatum* ATCC 25586, *S*. *mutans* Ingbritt.

### Applicability of the flow cytometry MDC assay

To validate the flow cytometry MDC assay further we synthesised three antimicrobial peptides with defined modes of antibacterial killing [11]; ovispirin (carpet mode), magainin II (toroidal pore mode) and alamethicin (barrel-stave mode) along with a potent lytic/pore forming AMP; caerin 1.1 [25] (S1 Table). The MIC, MBC and MDC of these peptides were then determined against each of the ESKAPE pathogens, MRSA and *E*. *coli*. Table 2 shows that each of the AMPs had different antimicrobial activities against each of the eight species tested. In comparing the activity of any one AMP, the flow cytometry MDC value was found to be similar/comparable to the MBC value and in the majority also had similar MIC values. Where MDC or MBC differed from MIC, the MIC was always significantly lower than the MDC or MBC reflecting that growth can be inhibited at a lower concentration than that required for cell lysis. The MIC value was observed to be significantly lower than the MDC or MBC values for; ovispirin with *E*. *aerogenes* and *K*. *pnuemoniae*; magainin II with *P*. *aeruginosa* and *E*. *coli*; alamethicin with *A*. *baumannii* and caerin 1.1 with *S*. *aureus* (MSSA and MRSA). In other experiments we found that *S*. *mutans* and *F*. *nucleatum* were susceptible to alamethicin and ovispirin and that the MDC value for each AMP was consistent with the MBC and MIC values (S2 Table).

**Table 2:** Comparison of the antimicrobial activity of three defined pore forming peptides and a membrane lytic peptide determined by microdilution growth assay (MIC), colony count assay (MBC) and the flow cytometry assay (MDC) for Gram positive and Gram negative bacteria.

		Gram-positive Bacteria			Gram-negative Bacteria				
Peptide	Activity (μM)^b^^,^ ^c^	*S*. *aureus*^d^ (MSSA)	*S*. *aureus*^d^ (MRSA)	*E*. *faecalis*^d^	*E*. *aerogenes*^d^	*P*. *aeruginosa*^d^	*K*. *pneumoniae*^d^	*A*. *baumannii*^d^	*E*. *coli*^d^
**Ovispirin (Carpet**^a^**)**	MIC	138.5±24.5	>250	78.8±40.7	45.9±13.8^e^	90.6±7.5	7.7±0.1^e^	2.6±1.1	9.6±3.9
	MBC	139.7±25.8	>250	72.4±13.7	135.2±26.3	95.9±9.8	21.7±3.7	2.0±1.0	8.5±0.7
	MDC	149.6±32.1	>250	75.8±5.3	120.3±15.6	94.5±12.7	32.1±2.3	3.6±1.4	10.9±3.1
**Magainin II (Toroidal pore**^a^**)**	MIC	>250	>250	>250	>250	26.1±1.2^e^	132.2±28.5	15.1±1.53^e^	29.5±3.7^e^
	MBC	>250	>250	>250	>250	56.3±4.2	142.3±19.5	20.0±1.0	48.1±12.4
	MDC	>250	>250	>250	>250	63.1±3.8	126.2±9.4	22.3±3.4	61.9±12.1
**Alamethicin (Barrel Stave**^a^**)**	MIC	31.4±7.4	50.8±3.5	30.1±9.6^e^	>250	>250	>250	142.9±19.6^e^	>250
	MBC	27.9±6.9	59.2±1.7	48.1 ± 5.4	>250	>250	>250	>250	>250
	MDC	30.3±8.2	-^f^	48.4±10.9	>250	>250	>250	>250	>250
**Caerin 1.1 (Lytic)**	MIC	5.5±0.2^e^	4.9±0.4^e^	27.1±15.0	40.0±8.2	39.3±21.4	14.6±5.1	3.1±0.4	8.45±0.2^e^
	MBC	9.9±2.3	13.9±2.4	17.5±10.6	51.1±6.3	51.4±27.1	20.0±7.1	3.0±1.0	15.6±0.4
	MDC	14.8±5.0	15.8	21.6±6.0	54.5±4.3	40.1±11.7	19.8±3.8	3.7±0.3	10.5

^a^Mechanism of pore formation [11].

^b^Activity expressed as μM is the average of 3 assays ± standard deviation.

^c^MIC, MBC and MDC determined following incubation of bacteria with peptide for 90 mins using standard protocols [24].

^d^Bacterial strains *S*. *aureus* (MSSA) ATCC 29213; *S*. *aureus* (MRSA) ATCC 43300; *E*. *faecalis* ATCC 29212; *E*. *aerogenes* ATCC 13048; *P*. *aeruginosa* strain PAO1-LAC, ATCC 47085; *K*. *pneumoniae* ATCC 13883; *A*. *baumannii* strain 2208, ATCC 19606; *E*. *coli* ATCC 25922.

^e^significantly different (p < 0.05) from MBC and MDC.

^f^no membrane disruption detected.

**Fig 2:**
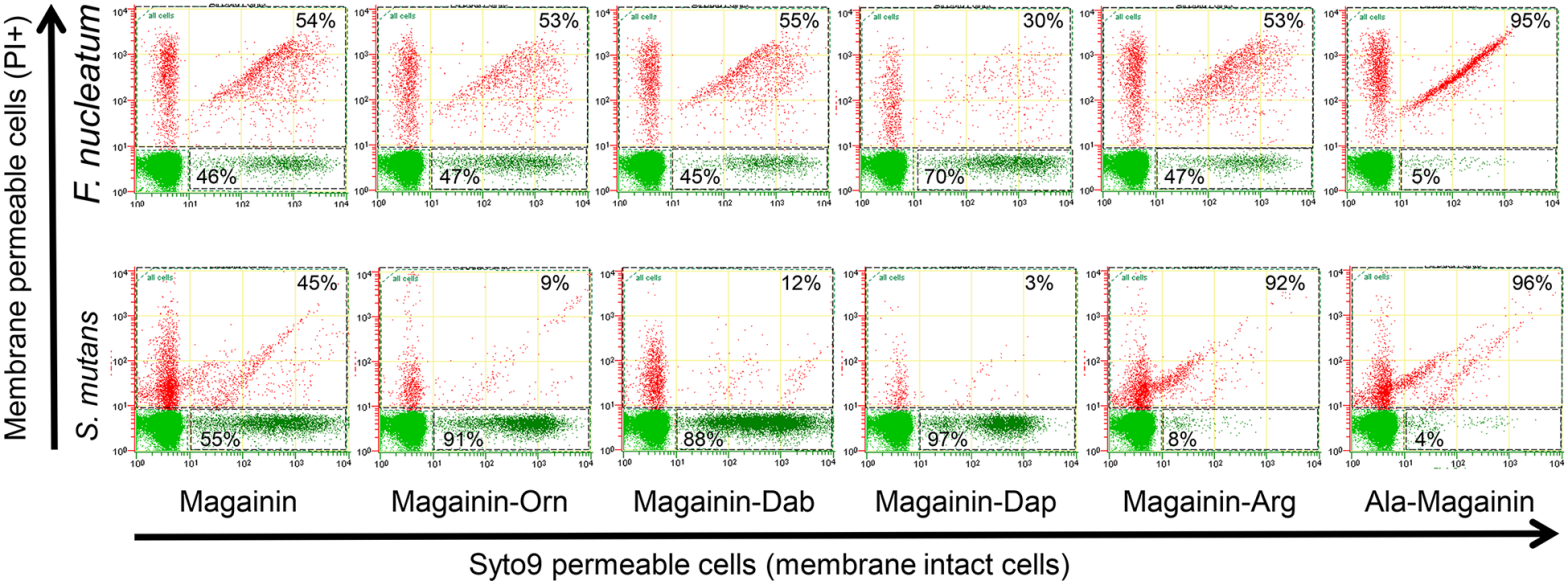
Flow cytometry dot plots of *F*. *nucleatum* and *S*. *mutans* incubated with magainin analogues at a concentration equivalent to the MIC50 concentration of magainin. Cells stained with Syto9 dye (membrane permeable) and propidium iodide dye (membrane impermeable).

A potential advantage of the flow cytometry assay is the speed at which AMP analogues can be screened in a semi or fully quantitative assay. To investigate this we synthesised five magainin II analogues with two critical properties for AMP activity altered (cationicity and α-helicity). Cationic properties were altered by substituting all of the lysine residues (residues 4, 10 11, 14) with either; arginine (Arg), ornithine (Orn), diaminobutyric acid (Dab) or diaminopropionic acid (Dap), and α-helicity increased by replacing residues 8 (Ser), 13 and 18 (Gly) with alanine. The magainin II sensitive bacteria, *S*. *mutans* and *F*. *nucleatum* were then screened in the flow cytometry assay with each of the analogues at a peptide concentration equivalent to MDC/MBC50 which equates to a 50:50 membrane intact:disrupted cell population (Fig 2). For *F*. *nucleatum* the Orn, Dab and Arg analogues did not alter the intact:disrupted ratio of 50:50, however the Dap analogue induced less disruption, while the Ala analogue increased disruption. For *S*. *mutans* the Orn, Dab and Dap analogues induced less disruption, while the Arg and Ala analogues increased disruption. To validate this semi-quantitative screening assay we then determined the MIC, MBC and MDC for each analogue (Table 3). The MIC, MBC and MDC values for each analogue reflected the screening assay, in that where membrane disruption was found to be similar, increased or decreased, the MIC, MBC and MDC values were either similar, lower or higher respectively when compared with that of native Magainin II (Table 3). Although the Arg and Ala analogues induced greater cell lysis compared with native magainin II they also induced greater hemolysis of red blood cells and had a lower therapeutic index (S3 Table). Interestingly for *F*. *nucleatum* the Dab analogue induced less hemolysis resulting in a higher therapeutic index compared with native magainin II (S3 Table).

**Table 3:** Antimicrobial activity of Magainin analogues against *S*. *mutans*, and *F*. *nucleatum*.

	*S*. *mutans* Ingbritt			*F*. *nucleatum* ATCC 25586		
Magainin II Analogue	MIC^a^^,^ ^b^ (μM)	MBC^a^^,^ ^b^ (μM)	MDC ^a^ (μM)	MIC^a^^,^ ^b^ (μM)	MBC^a^^,^ ^b^ (μM)	MDC ^a^ (μM)
Native	123.4±9.2	117.1±2.3	119.3±6.4	10.1±0.6	7.3±1.0	9.4±3.5
Orn	>200^c^	>200^c^	>200^c^	13.1±0.5	14.9±0.2	11.4±0.3
Dab	>200^c^	>200^c^	>200^c^	6.3+0.5	12.0+0.6	11.0±1.0
Dap	>200^c^	>200^c^	>200^c^	18.4±9.7^c^	29.7±0.4^c^	29.8±6.9^c^
Arg	41.8±6.8^c^	37.3±1.9^c^	42.5±5.7^c^	2.5±0.3^c^	2.5±0.8^c^	4.9±0.2^c^
Ala	7.1±0.7^c^	3.4±0.5^c^	7.3±0.4^c^	0.6±0.1^c^	0.4±0.1^c^	0.8±0.1^c^

^a^Activity expressed in μM is the average of 3 assays ± standard deviation.

^b^MIC and MBC determined following incubation of bacteria with peptide for 90 mins using standard protocols [24].

^c^Antimicrobial activity significantly different (p < 0.05) compared to Magainin activity (MIC, MBC or MDC).

## Discussion

The results presented here, although not exhaustive in terms of AMPs and bacterial species tested clearly illustrate the potential of this novel method in its ability to rapidly screen and identify lead antimicrobial compounds in a semi- or fully-quantitative assay. The data show that caerin 1.1 is effective against all of the ESKAPE pathogens and in particular against *A*. *baumannii* which is regarded as a significant emerging antibiotic resistant pathogen [1, 26]. We are the first to show that both ovispirin and caerin 1.1 are effective against *A*. *baumannii* and *E*. *aerogenes*. Further, our data corroborate earlier reports on the activity of the other AMPs tested and extend our knowledge on alamethicin being ineffective against Gram-negative bacteria, although it may have weak bacteriostatic activity against *A*. *baumannii* [11, 27–29].

Major advantages of the flow cytometry method compared to the traditional growth based assays are (a) the speed of quantifying antimicrobial activity of a peptide, (b) the method is applicable to all of the known lytic modes of AMP action, (c) rapid identification of chemical modifications that enhance or diminish AMP activity, (d) the ability to screen multi-species and/or multiple clinical isolates in a quantitative assay and (e) that the flow cytometry assay measures the direct effect of the AMP on cell membrane disruption/lysis, rather than cell survival as is determined by the growth assays. To determine MIC or MBC of an AMP, the traditional growth assays would take around 24 hours, while the flow cytometry method described here takes 3 hr. In combination with a hemolysis assay taking 2.5 hours it is now possible to more rapidly determine the therapeutic index and potential value of an AMP, thus reducing AMP development time. By using the MDC/MIC/MBC50 of the parent AMP for comparison AMP analogues can be rapidly screened using the flow cytometry method to determine the relative efficacy of each chemically modified analogue. Screening AMP analogues using MIC/MBC growth assays takes at least 2 days, whereas the flow cytometry method can be completed in only 7 hr. Furthermore, the flow cytometry method shows small positive and negative effects a modification has on AMP activity, which provides greater insight for further analogue development, which may be missed by the traditional growth assays.

The MDC values for all of the AMPs tested was found to be comparable to the corresponding MBC values, being consistent with MDC measuring cell lysis/death. This is further supported where the mode of action of the AMP was more bacteriostatic than bactericidal as was the case with ovispirin for *E*. *aerogenes*; magainin II for *E*. *coli* and alamethicin for *E*. *faecalis* and *A*. *baumannii*. A bacteriostatic AMP exhibits a lower MIC than MBC, and this was also the case compared with the MDC for these AMPs. When MIC = MBC the mode of antimicrobial action is defined as bactericidal and in our study where MIC and MBC were similar, MDC was also equivalent. However, an exception to this rule was seen in the activity of alamethicin against MRSA, which indicates that the flow cytometry method reveals more than simple bactericidal activity of an AMP. Alamethicin was effective against MSSA as well as MRSA, albeit at a lower MIC/MBC, but membrane disruption, as detected by PI inclusion (MDC), was observed for MSSA and not MRSA. This difference could be due to alamethicin’s mode of action which is classed as barrel-stave. Typically, alamethicin forms an 8–10 peptide bundle forming a 1.8 nm internal diameter pore. However, this has been shown to decrease to a 6-peptide bundle depending on the lipid membrane composition [30, 31]. The 6-peptide bundle has been shown to have a minimal internal pore size of 0.25 nm, which would exclude PI with a Stokes radius of 0.6 nm but still allow the unregulated flow of ions such as K^+^ having an ionic radius of 0.13 nm [30, 31]. The antibiotic methicillin is well known to induce lipid compositional changes in the membrane of MRSA compared to MSSA, and this could favour a 6-peptide bundle for MRSA [32]. Thus the flow cytometry method in conjunction with the growth based assays suggests that the alamethicin-peptide bundle/pore size may alter confirming the research using model lipid systems. The example of alamethicin and MRSA, does point to a limitation of the assay, that being an AMP must be lytic and induce a pore size greater than PI’s Stokes radius, however, few AMPs form pores less than 0.6 nm [11]. Alternatives to using PI to measure membrane disruption would be to use fluorophores that measure membrane potential. This potentially would have utility for AMPs such as proline-rich AMPs that typically act on internal targets and cause shifts in membrane potential but are not membranolytic.

The improvement in the sensitivity of forward and side scatter detectors in flow cytometers has resulted in a substantial increase in microbial flow cytometry. We and others have used flow cytometry to show variation in AMP pore size in bacteria [33], kinetics of AMP activity [34] as a method to determine antibiotic susceptibility [35] and that AMPs permeabilise bacteria [36, 37]. Thus, we anticipate that this AMP flow cytometry method would have wide ranging applicability. Furthermore it expedites AMP development by offering a high throughput, rapid and quantitative assay for a large number of synthetic AMPs against a wide range of bacteria.

## Materials and Methods

### Bacterial strains and growth conditions

The bacterial strains; *Fusobacterium nucleatum* ATCC 25586, *Streptococcus mutans* Ingbritt, *Staphylococcus aureus* (MSSA) ATCC 29213; *Staphylococcus aureus* (MRSA) ATCC 43300; *Enterococcus faecalis* ATCC 29212; *Enterobacter aerogenes* ATCC 13048; *Pseudomonas aeruginosa* strain PAO1-LAC, ATCC 47085; *Klebsiella pneumoniae* ATCC 13883; *Acinetobacter baumannii* strain 2208, ATCC 19606; *Escherichia coli* ATCC 25922 lyophilised or glycerol stocks were obtained from the culture collection of the Oral Health Cooperative Research Centre, The Melbourne Dental School, University of Melbourne, Australia. These strains were selected for this study as they are widely used in antimicrobial studies and are considered to be important pathogens and major targets for antibiotic resistant studies. Bacteria were grown aerobically and maintained by passage at ambient temperature (*E*. *coli*, *K*. *pneumoniae*, *A*. *baumannii* and *S*. *aureus* strains) or at 37°C (*P*. *aeruginosa*, *E*. *faecalis* and *E*. *aerogenes*) on horse blood agar (10% v/v defibrinated horse blood, 4.4% w/v Oxoid Blood Agar Base No. 2). *S*. *mutans* and *F*. *nucleatum* were grown anaerobically (MK3 anaerobic workstation, Don Whitley Scientific Limited, England) and maintained by passage at 37°C on Todd Hewitt agar (3.6% w/v Oxoid Todd-Hewitt Broth, 1.5% w/v sucrose, 1.5% w/v BactoTM Agar, 0.8% w/v Oxoid Yeast Extract) or horse blood agar, respectively. For the antimicrobial peptide assays a 20 mL starter culture was produced by taking single colonies from blood or Todd Hewitt agar plates, respectively, to inoculate either: Luria Broth (LB; 1% w/v BactoTM Tryptone, 1% w/v NaCl, 0.5% w/v Oxoid Yeast Extract, pH 7.5, Thermo Scientific Pty, Ltd, Sydney, Australia) for bacteria; *E*. *coli*, *K*. *pneumoniae*, *P*. *aeruginosa*, *A*. *baumannii*, *E*. *faecalis*, *E*. *aerogenes* and *S*. *aureus* which were grown aerobically at 37°C, or Todd Hewitt broth (3.6% w/v Oxoid Todd-Hewitt Broth, 1.5% w/v sucrose, 0.8% w/v Oxoid Yeast 9 Extract) for *S*. *mutans*, or Brain Heart Infusion broth (BHI; 3.7% w/v Oxoid BHI, 0.05% w/v L-cysteine) for *F*. *nucleatum* which were both grown anaerobically at 37°C. After overnight incubation, 0.5–2.0 mL of the starter culture was used to inoculate the appropriate fresh broth (200 mL) and growth monitored at 650 nm using a spectrophotometer (model 275E; Perkin-Elmer, Sydney, Australia) with culture purity checked by microscopic examination and culture. Bacteria were harvested during late exponential growth phase and viability and bacteria/mL determined using a BacLight viability kit (Invitrogen, Sydney, Australia) and a Quanta SC-MPL flow cytometer (Beckman coulter Pty, Ltd, Sydney, Australia).

### Synthesis of Antimicrobial Peptide (AMPs)

O-Benzotriazole-*N*,*N*,*N'*,*N'*-tetramethyluronium hexafluorophosphate (HBTU), 1hydroxybenzotriazole (HOBt), diisopropylethylamine (DIPEA), *N*,*N*-dimethylformamide (DMF), piperidine, trifluoroacetic acid (TFA) and 9-fluorenylmethoxycarbonyl (Fmoc) amino acids were obtained from Auspep Pty Ltd (Melbourne, Australia). Fmoc-Phenylalaninol was obtained from ChemPep Inc (FL). Triisopropylsilane (TIPS) was obtained from Aldrich (New South Wales, Australia). 1,8-diazabicyclo[5.4.0]undec-7-ene (DBU), 4-dimethylamino-pyridine (DMAP) and N,N’-diisopropyl-carbodiimide (DPCIDI) were obtained from Sigma Chemical Company (New South Wales, Australia). Diethyl ether and dichloromethane (DCM) were obtained from BDH (Poole, UK). diethylaminosulfur trifluoride was obtained from Alfa Aesar (WA). Unless otherwise stated chemicals were of peptide synthesis grade or its equivalent.

The antimicrobial peptides; caerin 1.1, melittin, ovispirin, magainin II and magainin II analogues (S2 Table) were chemically synthesized on a CEM Liberty microwave peptide synthesizer (Ai Scientific, Victoria, Australia). The peptide-resins were assembled from Fmoc-Rink-AM SURETM Resin or Fmoc-Gly-TGA resin (Merck Millipore Pty Ltd, NSW Australia) to produce the C-terminal carboxyamide or carboxylic acid AMPs, respectively, in the Fmoc/tBu mode of synthesis. For a 0.1 mmol reaction scale, Fmoc-deprotection was performed in two stages by initial treatment with 20% piperidine/0.1 M HOBt/DMF (v/v, 7 ml) under microwave radiation for 30 s (40 W, 40°C), followed by filtration and a second addition of the above solution (45 W, 75°C; 3 min). The peptide-resins were then rinsed with DMF (4 × 7 ml). Acylation, where required, was achieved by the addition of a solution containing amino acid (5 eq, relative to reaction scale), HBTU (5 eq) and DIEA (10 eq) in DMF/NMP (7:1, v/v; 4 ml) to the Nα-deprotected peptide-resin and the mixture agitated under microwave radiation for 10 min (30 W, 75°C, vessel under external chilled air flow). Dichloromethane (DCM) (5 × 2 min) was used to rinse the peptide-resins prior to the cleavage step. The peptide was cleaved from the resin support by the addition of TFA/TIPS/thioanisole/phenol/water (90:2.5:2.5:2.5:2.5, % v/v/v/v/v; 5 ml) for 2.5 h or 4 h for Arg containing AMPs, after which the cleavage filtrates were evaporated under nitrogen flow and the crude product was isolated by precipitation in cold ether (4 × 30 ml).

The crude peptides were purified using an Agilent 1200 series liquid chromatograph instrument (Agilent, NSW, Australia) equipped with a UV detector (model G1316A) and a Zorbax 300 SB-C18 reversed phase column (9.4 mm × 25 cm). Crude peptide analysis was achieved using a linear acetonitrile gradient in 0.1% TFA at a flow rate of 4 mL/min (linear gradient of 0 to 54% CH_3_CN over 15 min). Analysis of the purified peptide was performed using an Esquire HCT electrospray ionization-mass spectrometry system (Bruker Daltronics, NSW, Australia), each of the purified peptides gave the expected masses (S2 Table).

For the synthesis of Alamethicin, Fmoc-Phenylalaninol-Resin was produced by suspending 2-Chlorotrityl resin (750 mg; loading 1.36 mmol/g) in a solution of DCM and DMF [6ml; 1:3 (v/v)] followed by the addition of Fmoc-Phenylalaninol (545mg, 1.46 mmol) and pyridine (231 mg, 2.92 mmol) and the mixture gently stirred for 16 h, after which time methanol (4 ml) was added, the resin stirred for a further 2 h, filtered, rinsed with DCM and dried under reduced pressure overnight. Resin substitution was determined by incubating a resin sample (39.2 mg) in 2% DBU/DMF (2 mL) for 30 min. Acetonitrile was then added to achieve a final volume of 10 mL and the amount of Fmoc in solution determined by absorbance at 490nm and the resin substitution determined using control and reference solutions, which gave a resin loading of 0.31 mmol/g. The addition of Fmoc-aminoisobutyric acid (Fmoc-Aib) into the peptide chain was accomplished using Fmoc-Aib acid fluoride (Fmoc-Aib-F), whereby, Fmoc-Aib-F was synthesised by diethylaminosulfur trifluoride (DAST, 5.3 mL; 40.0 mmol) was added to a suspension containing Fmoc-Aib-OH (7.5 g; 30.7 mmol) in anhydrous DCM (30 mL) under nitrogen. After 1 h agitation, the reaction mixture was washed with cold water (2 ×40 mL) and the DCM layer collected and dried with Mg_2_SO_4_. The solution was then filtered, the solvent evaporated and the resultant light yellow powder re-dissolved in DCM (20 mL) and n-hexane (20 mL) and placed at 4°C for 48 h. The recrystallized product was filtered, washed with cold n-hexane and dried under reduced pressure. Three crystallizations were performed to give a final yield of 7.3g. Fmoc-Aib-F purity was determine by converting Fmoc-Aib-F to the methyl ester (Fmoc-Aib-OMe) by dissolving the crystalline product in methanol (4.75 mL) and pyridine (0.25 mL). After 10 min, DCM (10 mL) was added, the solution washed with 10% citric acid (2 × 10 mL) and the DCM layer evaporated. The resultant white product was dissolved in 60% buffer B containing a small amount of isopropanol and analyzed by RP-HPLC and ESI-MS which showed the presence of Fmoc-Aib-OMe in > 95% purity [m/z 340.0; (M+H)+]. The synthesis of alamethicin was performed using Fmoc-Phenylalaninol-Resin (359 mg; 0.1 mmol) and a CEM Liberty Microwave Synthesizer as described above for the other AMPs, with following modifications: Fmoc-Aib-F was coupled by the addition of the amino acid fluoride in DMF (5 eq, 2.5 mL) and DIEA in NMP (9 eq, 0.5 mL) to the peptide-resin followed by microwave heating (10 min, 30 W, 75°C). The peptide-resin was then filtered and the coupling step repeated once, followed by rinsing with DMF (3 × 7 mL). Upon completion of the final Fmoc deprotection step, the peptide-resin was rinsed with DMF (3 × 7 mL), DCM (3 × 10 mL) and dried under reduced pressure. A solution containing acetic anhydride (0.26 mL) and DIEA (0.98 mL) in DMF (4.0 mL) was then added and the mixture agitated for 30 min. Following final rinsing of the peptide-resin with DCM (5 × 10 mL), the resin-bound peptide was dried under reduced pressure and cleaved by the addition of TFA/DCM/H2O/TIPS (47:47:4:2; v/v; 2 h). The solvent was evaporated and the crude peptide precipitated in cold diethyl ether. Crude peptide was dissolved in 25% Buffer B/75% Buffer A and purified as described above using a linear gradient of 50–90% Buffer B in 40 min. The desired fraction was identified by MALDI-TOF MS using an Ultraflex TOF/TOF instrument (Bruker) in negative ion and reflectron mode.

### Antimicrobial assays

Antibacterial assays were undertaken to determinate the minimum inhibitory concentration (MIC), minimum bactericidal concentration (MBC) and minimum membrane disruptive concentration (MDC) of each of the antimicrobial peptides (AMP) and AMP analogues (S1 Table). For each bacterim a stock solution (2.5 × 10^6^ cells/mL) in the respective media was made and an aliquot incubated with AMPs within 15 minutes from viable cell count and stock preparation. All AMPs were dissolved in DMSO and a 500 μM stock solution prepared by adding the respective media and serial dilutions (250–0.244 μM) of the AMP in media (100 μL/well) made just prior addition of bacteria. DMSO was found to be necessary for solvating the AMPs in media and the final assay concentration of DMSO was ≤ 2.5% v/v, which did not affect bacteria viability or susceptibility to AMPs. One hundred microliter aliquots of the bacterial stock solution (2.5 × 10^5^ cells/well) were added to the AMP serial dilutions and incubated at 37°C for 90 min. Assay preparation and incubations were conducted in aerobic or anaerobic conditions dependent on the bacteria. Bacteria were also incubated in the absence of AMP to serve as a growth control for the assay. After the 90 minute incubation period the antimicrobial activity (MIC, MBC and MDC) was determined as follows;

For MIC the CLSI broth microdilution assay [16] was followed as we have previously described [33]. Briefly, after the 90 minute incubation, bacterial growth was monitored at 10 min intervals over a 12 or 24 hour period (bacteria dependent) at OD_650_ using an iEMS microplate reader (Pathtech Pty Ltd, Melbourne, Australia) which incubated the cultures at 37°C. The MIC was calculated using the Lambert and Pearson growth curve analysis method [38], by plotting the relative growth at each peptide concentration compared to maximal growth (determined as the point when bacteria incubated in media alone entered stationary phase of growth, 100% growth), the MIC was determined as the lowest peptide concentration (μM) required to completely inhibit the growth of the bacteria i.e the intersection of the linear curve with the x-axis.

For determination of MBC, the CLSI protocol [16] was followed, with the modifications being that due to the rapid action of the AMPs the assay incubation time was shortened from 16–20 hours to 90 minutes. Following incubation cells were pelleted by centrifugation (7,000g, 30 mins) and resuspended in 10 assay volumes (2 mL) of appropriate media and aliquots plated on agar and incubated for 24 to 48 h at 37°C (aerobically or anaerobically, bacteria dependent), and colony forming units (CFU) quantified. The MBC was thus determined as the lowest concentration (μM) of AMP yielding ≥ 99.9% reduction in the initial colony count after incubation.

For determination of MDC a 50 μL aliquot of the bacteria/AMP was mixed with 100 μL of 0.9% w/v saline containing 0.1% v/v of SYTO^®^ 9 (3.34mM stock solution) and 0.1% v/v of PI (50μg/mL stock solution) and incubated for 10 minutes at room temperature in the dark. Following incubation the bacterial cell samples were analysed using a Cell Lab Quanta SC MPL flow cytometer (Beckman Coulter) equipped with a 100 W stabilized mercury arc lamp with wavelengths of 365, 404, and 435 nm, and a 488 nm diode laser. The fluorescence from SYTO^®^ 9 was measured through a 525-nm band-pass filter (Fluorescent Channel 1, FL-1), and the red emission of PI was measured with a 670-nm long pass filter (Fluorescent Channel 3, FL-3). A minimum of 100,000 bacterial events were collected and the multi-parametric data analyzed using the Cell Lab Quanta SC software. NB for flow cytometers not fitted with an electrical volume detector for cell counting, the addition of microbeads to the samples can be used to determine cell numbers. MDC was calculated by plotting the percent PI+ cells for the each peptide concentration and fitting a linear curve to the data that is ≤ 99% PI+ cells and ≥ 1–5% PI+ cells (minimum of 4 data points). Inclusion of data points outside of these PI+ cell percentages will result in a positive or negative skewing of the MDC value. By solving the line equation (y = mx + C) for x (peptide concentration) when y = 1 (where 1 = 100% of PI+ cells) the MDC (x) can be determined i.e. MDC = x = (y − C)/m. The MDC was thus determined as the lowest concentration (μM) of AMP yielding ≥ 99.0% PI+ cells.

### Hemolysis assay

Fresh sheep red blood cells in Alsever’s solution (RBCs, Equicell, Victoria, Australia) were diluted 1 in 20 in PBS (pH 7.4), pelleted by centrifugation, and washed three times in PBS (1000 g, 10 min). The RBCs were counted using a cell counter (Coulter Particle Counter Z series, Beckman Coulter) and diluted to a final concentration of 2 × 10^7^ cells/mL. 100 μL aliquots of the RBC solution were seeded into a V-bottomed 96-well plate containing 100 μL of serial dilutions (250–0.244 μM) of the AMP in PBS and incubated in a humidified atmosphere containing 5% CO_2_ at 37°C for 2 h. Following incubation, the RBCs were pelleted centrifuged (1000 g, 10 min) and the amount of haemoglobin in 100 μL aliquots determined by measuring the absorbance at 405 nm using a microplate reader (PerkinElmer 1420 Multilabel Counter VICTOR3). Positive and negative controls for hemolysis were taken as RBC lysed with 0.5% Triton X-100 (1:1 v/v) and RBC suspension in PBS, respectively. The percentage of hemolysis was calculated using the following formula:

% Hemolysis = [(A405 test sample—A405 negative control) / (A405 positive control—A405 negative control)] × 100

The percentage hemolysis was plotted against peptide concentration and linear regression analysis was used to determine the hemolytic concentration needed to lyse 50% (HC50) of RBCs.

### Inhibition of cell proliferation assay

To determine the inhibition of cell proliferation, 100 μl of HEK-293 (ATCC CRL-1573TM) cells (5×10^3^) in media (Eagle's Minimum Essential Medium (EMEM) supplemented with 10% v/v foetal bovine serum) were seeded into 96-well plates containing 100 μL serial dilutions (250–0.244 μM) of the AMP in media and cultured at 37°C, 5% CO2 incubator. After 28 h, 20 μL of 3-(4,5-dimethylthiazol-2-yl)-5-(3-carboxymethoxyphenyl)-2-(4-sulfophenyl)-2H-tetrazolium (MTS) solution (CellTiter 96 AQueous Non-Radioactive Cell Proliferation Assay kit, Promega) were added to each well and the plates were incubated for a further 1–2 h at 37°C, 5% CO2 incubator. Cell proliferation was determined by measuring absorbance at 490 nm using a microplate reader (PerkinElmer 1420 Multilabel Counter VICTOR3). Positive and negative controls for inhibition were taken as HEK-293 cells incubated with mytomycin C (5 μg/mL, Sigma Pty Ltd) and HEK-293 cells incubated in media alone, respectively. The percentage of inhibition was calculated using the following formula:

% Inhibition = [(A490 test sample—A490 negative control) / (A490 positive control—A490 negative control)] × 100

The percentage of inhibition was plotted against peptide concentration and linear regression analysis was used to determine the AMP concentration needed to inhibit 50% (IC50) of the HEK-293 cells.

## Supporting Information

S1 Fig Flow cytometry dot plots of F. nucleatum incubated (90 mins) with the antimicrobial peptide melittin at 0.5x MIC/MBC (2μM) and 10x MIC/MBC (40μM) and membrane disrupted populations visualised using the optimal Syto9/PI concentrations of 3.34mM/50μg/mL, respectively. (DOC)

S2 Fig Increasing concentration of the antimicrobial peptide magainin II increases bacterial membrane permeability to propidium iodide. (DOC)

S1 Table Amino acid sequence of antimicrobial peptides. Standard single letter abbreviations for amino acids used.
(DOC)

S2 Table Comparison of the antimicrobial activity of three defined pore forming peptides determined by microdilution growth assay (MIC), colony count assay (MBC) and the flow cytometry assay (MDC) for Gram positive and Gram negative bacteria. (DOC)

S3 Table Therapeutic index of Magainin analogues. (DOC)
