# Supplementary material for: A Rapid and Quantitative Flow Cytometry Method for the Analysis of Membrane Disruptive Antimicrobial Activity
Source: PLoS One. 2016 Mar 17;11(3):e0151694. doi: 10.1371/journal.pone.0151694 (PMC4795541; doi:10.1371/journal.pone.0151694)
Supplement: S1 Fig — (DOC) [file pone.0151694.s001.doc]

**S1 Fig. Flow cytometry dot plots of *F. nucleatum* incubated (90 mins) with the antimicrobial peptide melittin at 0.5x MIC/MBC (2µM) and 10x MIC/MBC (40µM) and membrane disrupted populations visualised using the optimal Syto9/PI concentrations of 3.34mM/50µg/mL, respectively.** The figure shows the typical 3 distinct cell populations; membrane intact (Syto 9+/PI-); membrane disrupted (Syto 9+/PI+ and Syto 9-/PI+).
