# Supplementary material for: A Rapid and Quantitative Flow Cytometry Method for the Analysis of Membrane Disruptive Antimicrobial Activity
Source: PLoS One. 2016 Mar 17;11(3):e0151694. doi: 10.1371/journal.pone.0151694 (PMC4795541; doi:10.1371/journal.pone.0151694)
Supplement: S2 Fig — (DOC) [file pone.0151694.s002.doc]

**S2 Fig. Increasing concentration of the antimicrobial peptide magainin II increases bacterial membrane permeability to propidium iodide.** (a) Flow cytometry dot blots of *F. nucleatum* incubated with increasing concentrations of magainin II and stained with Syto9 dye (membrane permeable) and propidium iodide dye (membrane impermeable). (b) Percent propidium iodide positive *F. nucleatum* cells correlates with increasing peptide concentration. Insert show the reciprocal plot of percent membrane disrupted cells and peptide concentration to determine MDC.
