# Supplementary material for: A Rapid and Quantitative Flow Cytometry Method for the Analysis of Membrane Disruptive Antimicrobial Activity
Source: PLoS One. 2016 Mar 17;11(3):e0151694. doi: 10.1371/journal.pone.0151694 (PMC4795541; doi:10.1371/journal.pone.0151694)
Supplement: S1 Table — Standard single letter abbreviations for amino acids used. (DOC) [file pone.0151694.s003.doc]

**S1 Table. Amino acid sequence of antimicrobial peptides. Standard single letter abbreviations for amino acids used.**

| Peptide | Amino acid sequence (single letter code) | Peptide masses (m/z) | |
| --- | --- | --- | --- |
|  |  | Calculated | Observed |
| Caerin 1.1 | GLLSVLGSVAKHVLPHVVPVIAEHL-CONH_2_ | 2584.2 | 2584.0 |
| Melittin | GIGAVLKVLTTGLPALISWIKRKRQQ-CONH_2_ | 2846.5 | 2846.7 |
| Ovispirin | KNLRRIIRKIIHIIKKYG-COOH | 2262.8 | 2262.6 |
| Alamethicin^a^ | XPXAXAQXVXGLXPVXXEQF-OH | 1963.2 | 1963.2 (-ve mode) |
| Magainin II | GIGKFLHSAKKFGKAFVGEIMNS-CONH_2_ | 2465.9 | 2465.6 |
| Magainin II – Lys analogue^b^ | GIGXFLHSAXXFGXAFVGEIMNS-CONH_2_ | 2405.8 | 2406.2 (x = Orn) |
|  |  | 2353.6 | 2354.2 (x = Dab) |
|  |  | 2297.6 | 2298.1 (x = Dpr) |
|  |  | 2578.0 | 2578.3 (x = Arg) |
| Magainin II – Ala analogue^c^ | GIGKFLHAAKKFAKAFVAEIMNS-CONH_2_ | 2478.0 | 2478.4 |

a = alamethicin is a C terminal alcohol and x = 2-Aminoisobutyric acid.

b = x = Ornithine (Orn), Diaminobutyric acid (Dab), Diaminoproponic acid (Dpr), Arginine (Arg).

c = underlined A (Alanine) indicates the placement of the ala substitution.
