# Supplementary material for: A Rapid and Quantitative Flow Cytometry Method for the Analysis of Membrane Disruptive Antimicrobial Activity
Source: PLoS One. 2016 Mar 17;11(3):e0151694. doi: 10.1371/journal.pone.0151694 (PMC4795541; doi:10.1371/journal.pone.0151694)
Supplement: S2 Table — (DOC) [file pone.0151694.s004.doc]

**S2 Table. Comparison of the antimicrobial activity of three defined pore forming peptides determined by microdilution growth assay (MIC), colony count assay (MBC) and the flow cytometry assay (MDC) for Gram positive and Gram negative bacteria.**

|  | Peptide | Activity (μM) ^b, c^ | *S. mutans*^d^ | *F. nucleatum*^d^ |
| --- | --- | --- | --- | --- |
| Barrel Stave^a^ | Alamethicin | MIC | 29.4 ± 8.2 | 22.6 ± 12.7 |
|  |  | MBC | 39.8 ± 0.3 | 18.0 ± 3.9 |
|  |  | MDC | 41.9 ± 8.3 | 21.1 ± 4.1 |
| Torroidal pore^a^ | Magainin II | MIC | 109.3 ± 20.4 | 10.2 ± 1.5 |
|  |  | MBC | 109 ± 20.2 | 8.1 ± 1.3 |
|  |  | MDC | 108.7 ± 16.7 | 9.5 ± 2.4 |
| Carpet^a^ | Ovispirin | MIC | 16.5 ± 1.7 | 4.2 ± 2.9 |
|  |  | MBC | 11.3 ± 5.3 | 4.7 ± 3.5 |
|  |  | MDC | 15.3 ± 2.7 | 4.6 ± 3.2 |

a = Mechanism of pore formation .

b = Activity expressed as μM is the average of 3 assays ± standard deviation.

c = MIC, MBC and MDC determined following incubation of bacteria with peptide for 90 mins using standard protocols.

d = Bacterial strains *F. nucleatum* ATCC 25586, *S. mutans* Ingbritt.
