# Supplementary material for: A Rapid and Quantitative Flow Cytometry Method for the Analysis of Membrane Disruptive Antimicrobial Activity
Source: PLoS One. 2016 Mar 17;11(3):e0151694. doi: 10.1371/journal.pone.0151694 (PMC4795541; doi:10.1371/journal.pone.0151694)
Supplement: S3 Table — (DOC) [file pone.0151694.s005.doc]

**S3 Table. Therapeutic index of Magainin analogues.**

| Peptide | Antimicrobial activity^a^ (MDC_50_, μM)^b^ | Hemolysis. (HC_50_, μM)^b^ | Therapeutic Index (hemolysis)^c^ | Inhibition of proliferation (IC_50_ μM)^b^ | Therapeutic Index (proliferation)^c^ |
| --- | --- | --- | --- | --- | --- |
| Magainin | 4.7±1.8 | 479.5±42.9 | 111.8 : 1 | 95.2±32.2 | 20.2 : 1 |
| Magainin-Orn | 5.7±0.2 | 721.1±65.3 | 126.5 : 1 | 167.5±42.1 | 29.4 : 1 |
| Magainin-Dab | 5.5±0.5 | >1000 | >182 : 1 | 178.1±39.4 | 32.4 : 1 |
| Magainin-Dap | 14.9±3.5 | >1000 | >67 : 1 | 285.1±19.4 | 19.1 : 1 |
| Magainin-Arg | 2.5±0.1 | 251.9±48.2 | 100.8 : 1 | 64.5±3.0 | 25.8 : 1 |
| Ala-Magainin | 0.4±0.1 | 9.6±0.9 | 24 : 1 | 5.6±1.4 | 14.0 : 1 |

a = antimicrobial activity against *F. nucleatum*.

b = Maximum peptide concentration = 1000 μM.

c = Therapeutic index determined for hemolysis by HC_50_/MDC_50_ or for inhibition of proliferation by IC_50_/MDC_50_.
